# Systemic immune-inflammation index predicts postoperative delirium in elderly patients after surgery: a retrospective cohort study

**DOI:** 10.1186/s12877-022-03418-4

**Published:** 2022-09-05

**Authors:** Yuxiang Song, Yungen Luo, Faqiang Zhang, Yulong Ma, Jingsheng Lou, Hao Li, Yanhong Liu, Weidong Mi, Jiangbei Cao

**Affiliations:** 1 Department of Anesthesiology, The First Medical Center of Chinese, PLA General Hospital, Beijing, China; 2 Medical School of Chinese People’s Liberation Army, Beijing, China

**Keywords:** Systemic-immune-inflammation (SII), Postoperative Delirium (POD), Inflammation, Elderly Patients, Biomarker

## Abstract

**Background:**

Postoperative delirium (POD) is a common complication among elderly patients after surgery. It is unclear whether the systemic immune-inflammation index (SII) can be a predictor of POD. We explored the prognostic value of the SII in predicting POD in elderly patients undergoing non-neurosurgery and non-cardiac surgery in a large retrospective cohort.

**Methods:**

We enrolled elderly patients undergoing non-neurosurgery and non-cardiac surgery between January 2014 and August 2019. Univariate and multivariate logistic regression analyses were performed to explore the correlation between POD and the SII value as both a continuous and categorical variable. Then, propensity score matching (PSM) analysis was applied to eliminate the confounding effect of covariates and prove our results. Subgroup analyses were then performed to discover the association between the SII and POD in different subgroups.

**Results:**

A total of 29,608 patients with a median age of 70 years (IQR: 67–74) were enrolled in the retrospective cohort. The cut-off value of the SII was 650, which was determined by the receiver operating characteristic (ROC) curve. The ORs of an SII value > 650 was 2.709 (95% CI:2.373–3.092, *P* < 0.001), 1.615 (95% CI:1.384–1.882, *P* < 0.001), 1.855 (95% CI:1.602–2.146, *P* < 0.001), and 1.302 (95% CI:1.106–1.531, *P* = 0.001) for prediction of POD in univariate model and three multivariate regression models. After PSM, the OR of an SII value > 650 was 1.301 (95% CI: 1.062–1.598, *P* = 0.011). The subgroup analysis indicated that the SII indicates a significantly increased risk of POD in patients with Hb < 130 g/L, 4*10^9^/L < WBC ≤ 10*10^9^/L, albumin < 39 g/L, or duration of MAP < 60 mmHg ≥ 5 min. The SII was found to be a useful prognostic predictor of POD for patients of different ages, sexes, and ASA classifications.

**Conclusions:**

The SII had a predictive value for POD in patients undergoing non-neurosurgery and non-cardiac surgery. As an index generated from routine blood tests, the SII has advantages regarding cost and time. After further validation, the SII may provide a new option for POD prediction.

**Supplementary Information:**

The online version contains supplementary material available at 10.1186/s12877-022-03418-4.

## Background

Postoperative delirium (POD) is a common complication of elderly patients after surgery and is defined as a reversible disturbance in attention, awareness, and cognition. Its incidence is 3 to 50% for different surgeries [1, 2]. Loss of independence, increased morbidity and mortality, and high health costs are common adverse effects of POD [2, 3]. Thirty percent of patients with delirium can benefit from multifactorial preventive measures and treatments if they can be identified before POD occurs [4]. Therefore, identifying and managing high-risk POD patients is critical in improving the outcomes of elderly patients after surgery.

Inflammation plays a critical role in the pathophysiology of POD [5, 6]. Several inflammatory biomarkers in serum or cerebrospinal fluid are associated with POD after different surgeries [7–10]. Compared with IL-6, IL-8, C-reactive protein, and S-100β, white-cell derived inflammatory biomarkers, namely, the neutrophil–lymphocyte ratio (NLR), platelet-to-lymphocyte ratio (PLR), platelet-to-white cell ratio (PWR), and systemic immune inflammation index (SII, platelet × neutrophil/lymphocyte), can be obtained for a lower cost and require less time. Some studies have shown the potential value of the NLR, PLR, and PWR in predicting POD after lower limb, head and neck free-flap reconstruction, total hip arthroplasty, abdominal surgery, oesophagectomy carotid endarterectomy, and cardiac surgery [11–15]. As a white-cell derived inflammatory biomarker, systemic immune inflammation index (SII) has been used to predict ischemic stroke, delayed vasospasm, microvascular dysfunction, and acute kidney injury [16–19]. However, the SII has not been applied to predict POD in elderly patients after surgery.

In this study, we explored the prognostic value of the SII in predicting POD in elderly patients after non-neurosurgery and non-cardiac surgery in a large retrospective cohort. The hypothesis is that an elevated SII value before surgery may indicate an increased risk of POD.

## Method

### Study design and patients

This study was a retrospective cohort study. All the perioperative data were obtained from patients at the first medical center of the Chinese PLA General Hospital from January 2014 to April 2019. Inclusion criteria: 1) patients ≥ 65 years and 2) patients undergoing non-neurosurgery and non-cardiac surgery with anesthesia. The exclusion criteria were as follows: 1) patients undergoing digestive endoscopy; 2) missing data on white cell count, neutrophils, lymphocytes, and platelets; and 3) missing data for over 50% of all variables. The study was conducted following the Declaration of Helsinki. The study was approved by the Ethics Committee Board of the First Medical Center of Chinese PLA General Hospital (No. S2019-311–03). Patient consent was waived because the study was retrospective, and all data were anonymized before analysis.

### Data collection

The dataset was established from the medical record system. For more accurate and broader clinical use of the models, we included both preoperative and intraoperative parameters of the models, which might be closely associated with POD, including the following aspects:

1. We collected the relevant patient demographics, including age; sex; body mass index (BMI); smoking; alcohol consumption; a combination of hypertension, diabetes, cardiovascular diseases and chronic obstructive pulmonary disease (COPD); chronic kidney disease (CKD); cerebrovascular disease; depression and anxiety; non-independent functional status; American Society of Anesthesiologists physical score (ASA); and days in the hospital before surgery.
2. The medication prescribed before surgery included nonsteroidal anti-inflammatory drugs (loxoprofen, acetaminophen, ibuprofen, celecoxib, meloxicam, diclofenac, etoricoxib, nimesulide), anticholinergic drugs (atropine, penehyclidine hydrochloride, scopolamine), benzodiazepines (midazolam, estazolam, diazepam, lorazepam, alprazolam, zolpidem, zopiclone), and antipsychotic drugs (quetiapine, olanzapine, droperidol, haloperidol, risperidone).
3. Laboratory tests (the most recent before surgery) were hemoglobin (Hb), WBC count, neutrophils, lymphocytes, monocytes, platelets, glucose (Glu), serum albumin, serum creatinine (Cre), alanine aminotransferase (ALT), aspartate aminotransferase (AST), total bilirubin, and prothrombin time (PT). SII = platelet × neutrophil/lymphocyte.
4. The intraoperative data included type of surgery, type of anesthesia, emergency status, duration of surgery and anesthesia, urine, blood loss, crystalloid, colloid, blood transfusion (red blood cells, whole blood, plasma, platelet, and cryoprecipitate, autologous blood, fibrinogen, albumin), use of glucocorticoids (dexamethasone, methylprednisolone), use of dexmedetomidine, duration of systolic blood pressure > 140 mmHg, and duration of MAP < 60 mmHg.

  

### Definitions of outcomes

The primary outcome was the incidence of POD within seven days postoperatively. It was captured through descriptive words documented in the medical records and confirmed by the neurologist. The inclusion criteria were as follows: 1) the postoperative medical records contained "mental status change", "confusion", "disorientation", "agitation", "delirium", "inappropriate behaviour", "inattention", "hallucinations", and "combative behaviour" [20] and 2) the postoperative drug regimen contained "quetiapine", "olanzapine", "haloperidol", "haloperidol", and "risperidone". The exclusion criteria were 1) preoperative medical records containing the “symptoms” mentioned above and 2) a preoperative drug regimen containing the “drugs” mentioned above. Neurologists rechecked all the delirium patients’ medical records diagnosing the POD using Diagnostic and Statistical Manual of Mental Disorders, fourth edition (DSM-IV) criteria [13].

### Correlation between the SII and POD

We divided the SII into multiple categories (using quartiles). The receiver operating characteristic (ROC) curve was also calculated to discover the optimal cut-off value of the SII in predicting POD. Then, we performed univariate and multivariate logistic analyses to explore the correlation between POD and the SII as continuous and categorical variables (with quartiles and optimal cut-off value). In multivariate logistic regression analysis, model 1 included the baseline characteristics (age, sex, BMI, smoking, alcohol consumption, hypertension, diabetes, cardiovascular disease, COPD, cerebrovascular disease, CKD, depression and anxiety, non-independent functional status, ASA), preoperative medications (anticholinergic drugs, NSAIDs, benzodiazepines, antipsychotic drugs), and preoperative laboratory tests (hemoglobin, WBC count, Glu, albumin, Cre, AST, ALT, PT). Model 1 adjusted for age, sex, BMI, ASA, alcohol, cerebrovascular disease, COPD, depression and anxiety, antipsychotic drugs, benzodiazepines, hemoglobin, WBC count, albumin, AST, ALT, PT, types of surgery, and anesthesia methods. Model 2 included intraoperative variables (emergency surgery, types of surgery, anesthesia methods, duration of surgery and anesthesia, blood loss, urine, crystalloid, colloid, blood transfusion, duration of SBP > 140 mmHg, duration of MAP < 60 mmHg) and intraoperative medication. Emergency surgery, duration of anesthesia, colloid, blood transfusion, duration of SBP > 140 mmHg, and MAP < 60 mmHg were adjusted in model 2. Model 3 included baseline characteristics, preoperative medications, preoperative laboratory tests, intraoperative variables, and intraoperative medication and adjusted for variables adjusted in model 1 and model 2.

### Propensity score-matching analysis and adjustment

Propensity scores are an alternative method for estimating the effect of receiving treatment when the random assignment of treatments to subjects is not feasible. Propensity score matching (PSM) refers to the pairing of treatment and control subjects with similar values on the propensity score and possibly other covariates and discarding all unmatched subjects [21]. The propensity score was computed based on the probability of patients having different levels of the SII and derived from synthesized baseline parameters using the multivariate logistic regression model. After propensity score generation, patients with different categorical SII values were randomly matched at a 1:1 ratio using the greedy nearest neighbor matching approach (maximum calliper width, 0.1). Kernel density plots of the propensity scores were applied to examine the equivalence between the matched patients. The standardized mean difference (SMD) was reported and used to evaluate the baseline differences between the two groups, where a value < 0.1 indicated minor differences.

### Subgroup analyses

After logistic regression analysis of different models and PSM analysis, subgroup analysis was conducted to explore the correlation between the SII and POD in different ages, sexes, ASA classification, hemoglobin, WBC count, albumin, duration of MAP < 60 mmHg subgroups. A forest plot was used to summarize the prediction of POD by the SII in subgroups.

### Statistical analysis

Normally distributed continuous variables are expressed as the mean (SD) and were compared using Student’s t test. If the continuous data are not normally distributed, they are shown as the median and interquartile range (IQR) and were compared with the Mann–Whitney test. Categorical variables are expressed as frequencies or percentages and were compared using the χ2 test or Fisher’s exact test. Statistical significance was accepted at the 0.05 level, and all tests were two-tailed. The logistic regression model was performed using R 4.0.1 (R Foundation for Statistical Computing, Vienna, Austria).

## Results

### Patient characteristics

**Fig. 1:**
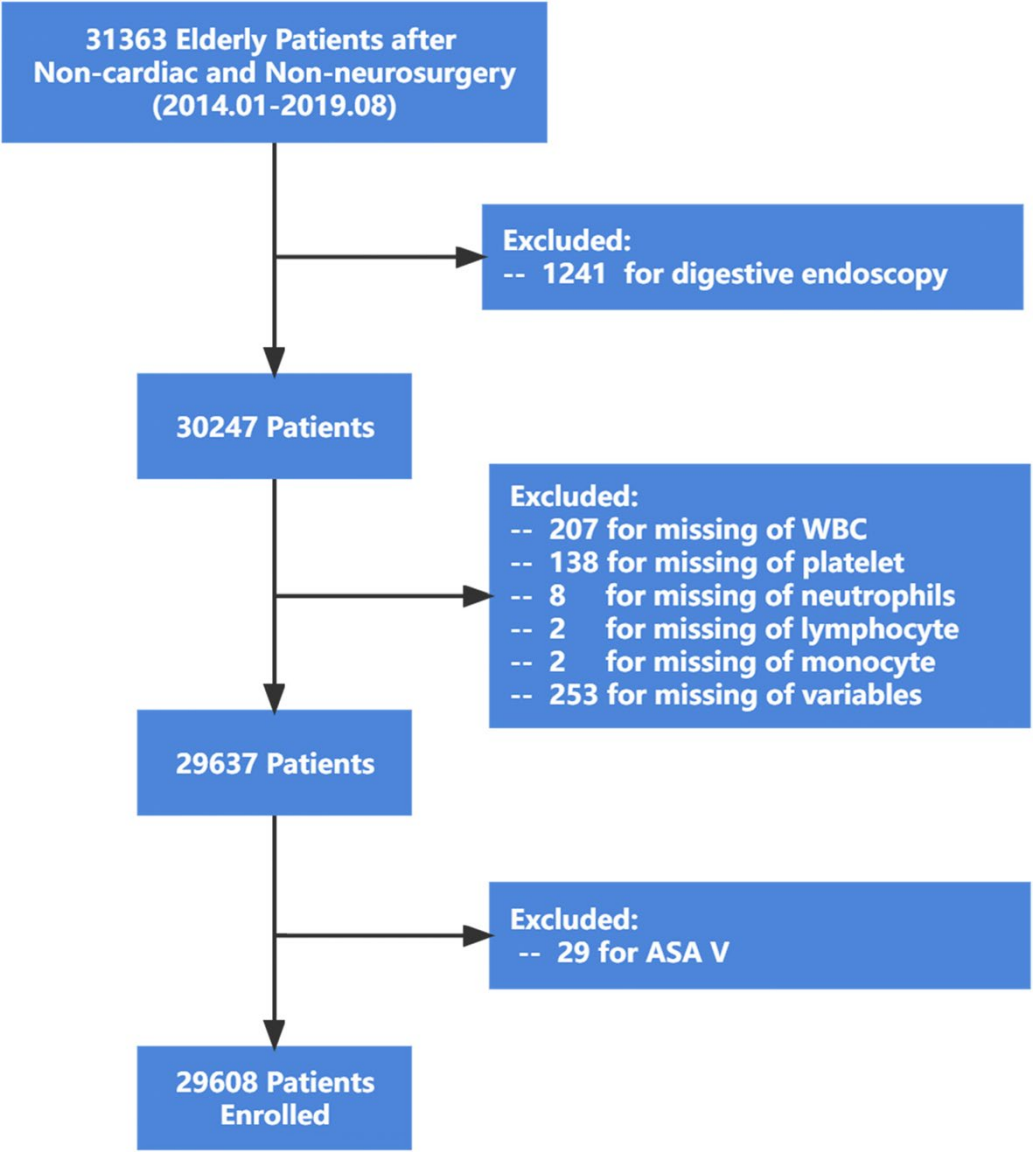
The enrolment flow chart of patients. ASA, American Society of Anesthesiologists classification; WBC, white blood cell

The medical records of 31,066 patients over 65 years old after non-neurosurgery and non-cardiac surgery from January 2014 to August 2019 at the First Medical Center of Chinese PLA General Hospital were retrospectively analyzed. After excluding 1116 patients for digestive endoscopy, 610 patients for missing variables, and 29 patients for ASA V, 29,608 patients were enrolled in the final analysis (Fig. 1). The median age was 70 years (IQR: 67, 74), and 50.9% of the enrolled patients (15,066/29608) were male. The median levels of hemoglobin, WBC count, and albumin were 130 (IQR: 119–141) g/L, 5.93 (IQR: 4.95–7.17) × 10^9^, and 39.8 (IQR: 37.3–42.3) g/L, respectively. The incidence of POD was 3.1% (927/29608) in the overall cohort.

The median SII value was 416 (IQR: 289–630) × 10^9^. The ROC curve showed that the optimal cut-off value of the SII in predicting POD was 650 × 10^9^, with an area under the ROC curve (AUC) of 0.63 (Additional file Figure A1). The baseline characteristics of the patients and the preoperative and intraoperative variables are shown according to SII ≤ 650 (*n* = 22,635) and SII > 650 (*n* = 6973). Of the baseline characteristics, except for cardiovascular disease, depression and anxiety, and the use of NSAIDs, all variables differed between the two groups. The patients in the SII > 650 group had more comorbidities (hypertension, diabetes, cerebrovascular disease, CKD, COPD) and longer stays in the hospital before surgery than those in the SII ≤ 650 group. The preoperative hemoglobin, WBC count, Glu, albumin, Cre, and PT also differed between the two groups. The difference was also shown in the intraoperative variables, such as type and duration of surgery and anesthesia, and fluid balance (blood loss, urine, blood transfusion, colloid, and crystalloid) (Table 1).

**Table 1:** The characteristics of patients before and after PSM

Characteristic	Before PSM (*n* = 29,608)			After PSM (1:1) (*n* = 10,062)		
	**SII ≤ 650 (*n* = 22,635)**	**SII > 650 (*n* = 6973)**	**SMD**	**SII ≤ 650 (*n* = 5031)**	**SII > 650 (*n* = 5031)**	**SMD**
**Age, years**	70(67,74)	71(67,76)	0.238	71.0 (67.0, 76.0)	71.0 (67.0, 75.0)	0.04
**Sex (male), n (%)**	11,208(49.5)	3858(55.3)	0.117	2716 (54.0)	2725 (54.2)	0.004
**BMI, kg·m**^**,2**^	24.62(22.45,26.94)	23.72(21.34,26.16)	0.261	24.0 (21.8, 26.4)	23.9 (21.6, 26.4)	0.024
**Smoking, n (%)**	5134(22.7)	1763(25.3)	0.061	1253 (24.9)	1268 (25.2)	0.007
**Alcohol consumption, n (%)**	4806(21.2)	1624(23.3)	0.049	1141 (22.7)	1185 (23.6)	0.021
**Hypertension, n (%)**	10,762(47.5)	3474(49.8)	0.046	2388 (47.5)	2499 (49.7)	0.044
**Diabetes, n (%)**	5245(23.2)	1761(25.3)	0.049	1283 (25.5)	1231 (24.5)	0.024
**Cardiovascular diseases, n (%)**	2209(9.8)	674(9.7)	0.003	517 (10.3)	473 (9.4)	0.029
**COPD, n (%)**	803(3.5)	382(5.5)	0.093	229 (4.6)	257 (5.1)	0.026
**Cerebrovascular disease, n (%)**	2114(9.3)	783(11.2)	0.062	506 (10.1)	542 (10.8)	0.023
**CKD, n (%)**	220(1)	142(2)	0.088	76 (1.5)	70 (1.4)	0.01
**Depression and anxiety, n (%)**	115(0.5)	46(0.7)	0.02	33 (0.7)	36 (0.7)	0.007c
**Non-independent functional status, n (%)**	5630(24.9)	3147(45.1)	0.435	2005 (39.9)	1867 (37.1)	0.056
**ASA, n (%)**			0.29			0.096
**I**	298(1.3)	59(0.8)		49 (1.0)	48 (1.0)	
**II**	18,466(81.6)	4963(71.2)		3862 (76.8)	3729 (74.1)	
**III**	3802(16.8)	1801(25.8)		1088 (21.6)	1179 (23.4)	
**IV**	69(0.3)	150(2.2)		32 (0.6)	75 (1.5)	
**Emergency surgery, n (%)**	348(1.5)	454(6.5)	0.255	147 (2.9)	193 (3.8)	0.051
**Hemoglobin, g·L**^**−1**^	132(122,142)	123(109,137)	0.5	127.0 (115.0, 137.0)	126.0 (113.0, 139.0)	0.014
WBC count, *10^**9**^**/L**	5.63(4.75,6.62)	7.4(6.08,9)	0.843	6.8 (5.7, 8.0)	6.7 (5.7, 7.9)	0.009
Monocytes, *10^**9**^**/L**	0.07(0.06,0.08)	0.06(0.05,0.08)	0.066			
**Glu, mmol/L**	5.05(4.63,5.73)	5.46(4.82,6.61)	0.339	5.2 (4.7, 6.2)	5.3 (4.8, 6.3)	0.017
**Albumin, g/L**	40.2(37.9,42.5)	38.1(34.7,41.3)	0.519	39.0 (36.4, 41.6)	38.9 (35.8, 42.0)	0.018
**Cre, μmol/L**	70.9(60.7,82.6)	70.1(58.8,83.6)	0.048	72.0 (61.1, 84.4)	70.40 (59.3, 83.0)	0.076
**Total bilirubin, μmol/L**	11(8.5,14.3)	10.8(7.9,15.7)	0.262	10.50 (8.0, 14.3)	10.70 (7.8, 14.9)	0.06
**AST, U/L**	14.7(11,21.1)	14.8(10.3,24.8)	0.174	14.9 (10.7, 22.3)	14.20(10.2, 23.1)	0.019
**ALT, U/L**	16.8(14.1,20.9)	16.7(13.4,23.3)	0.167	16.8 (13.9, 21.9)	16.4 (13.4, 21.8)	0.006
**PT, s**	13.2(12.7,13.7)	13.4(12.9,14.1)	0.237	13.3 (12.8, 13.9)	13.3 (12.8, 13.9)	0.035
**Preoperative medication, n (%)**						
**Anticholinergic drug**	13,292 (58.7)	3632 (52.1)	0.134	2807 (55.8)	2809 (55.8)	0.001
**NSAIDs**	1509 (6.7)	506 (7.3)	0.023	358 (7.1)	331 (6.6)	0.021
**Benzodiazepines**	5144 (22.7)	1493 (21.4)	0.032	1126 (22.4)	1051 (20.9)	0.036
**Antipsychotic drugs**	60 (0.3)	47 (0.7)	0.06	20 (0.4)	27 (0.5)	0.02
**Type of surgery, n (%)**			0.261			0.16
**Hepatopancreatobiliary and gastrointestinal surgery**	7265(32.1)	2849(40.9)		1932 (38.4)	2199 (43.7)	
**Orthopedic surgery**	6466(28.6)	2183(31.3)		1594 (31.7)	1367 (27.2)	
**Urinary surgery**	2238(9.9)	437(6.3)		352 (7.0)	308 (6.1)	
**Thoracic surgery**	1723(7.6)	415(6)		341 (6.8)	291 (5.8)	
**E.N.T**	1181(5.2)	301(4.3)		201 (4.0)	289 (5.7)	
**Gynecology**	839(3.7)	163(2.3)		134 (2.7)	157 (3.1)	
**Vascular surgery**	952(4.2)	229(3.3)		163 (3.2)	131 (2.6)	
**Others**	1971(8.7)	396(5.7)		314 (6.2)	289 (5.7)	
**Anesthesia method, n (%)**			0.134			0.056
**Basal anesthesia**	255(1.1)	121(1.7)		58 (1.2)	73 (1.5)	
**Epidural anesthesia**	426(1.9)	114(1.6)		94 (1.9)	63 (1.3)	
**Nerve blocks**	418(1.8)	205(2.9)		116 (2.3)	117 (2.3)	
**General anesthesia**	19,006(84)	5540(79.4)		4163 (82.7)	4169 (82.9)	
**General anesthesia combined with other anesthesia**	2530(11.2)	993(14.2)		600 (11.9)	609 (12.1)	
**Duration of surgery, min**	130.0(85.0,190.0)	140.0(95.0,210.0)	0.164	10.00 (0.00, 30.00)	10.00 (0.00, 30.00)	0.022
**Duration of anesthesia, min**	178.0(127.0,240.0)	190.0(140.0,260.0)	0.169	5.00 (0.00, 15.00)	5.00 (0.00, 15.00)	0.007
**Blood loss, ml**	100.0(30.0,200.0)	100.0(50.0,200.0)	0.113	100.0 (50.0, 200.0)	100.0 (50.0, 200.0)	0.033
**Urine, ml**	200.0(100.0,500.0)	250.0(100.0,500.0)	0.047	300.0 (100.0,600.0)	250.0 (100.0, 500.0)	0.063
**Crystalloid, ml**	1300.0(1100.0,2100.0)	1600.0(1100.0,2100.0)	0.153	1600.0 (1100.0, 2100.0)	1600.0 (1100.0, 2100.0)	0.055
**Colloid, ml**	500.0(0,500.0)	500.0(0,500.0)	0.163	500.0 (0, 500.0)	500.0 (0.0, 500.0)	0.063
**Blood transfusion, n**	2763 (12.2)	1420 (20.4)	0.222	821 (16.3)	949 (18.9)	0.067
**Duration of SBP > 140 mmHg, min**	5.0(0,25.0)	10.0(0,30.0)	0.141	10.0 (0, 30.0)	10.0 (0.0, 30.0)	0.022
**Duration of MAP < 60 mmHg, min**	5.0(0,10.0)	5.0(0,15.0)	0.104	5.0 (0, 15.0)	5.0 (0.0, 15.0)	0.007
**Intraoperative medication, n (%)**						
**Glucocorticoid**	14,303 (63.2)	4324 (62.0)	0.024	3155 (62.7)	3251 (64.6)	0.04
**Dexmedetomidine**	2262 (10.0)	692 (9.9)	0.002	532 (10.6)	442 (8.8)	0.061
**POD, n (%)**	514 (2.3)	413 (5.9)	0.185	170 (3.4)	219 (4.4)	0.051

*Abbreviations: SII* Systemic-immune-inflammation index, *SMD* Standardized mean difference, *PSM* Propensity Score Matching, *BMI* Body mass index, *COPD* Chronic obstructive pulmonary disease, *CKD* Chronic kidney disease, *ASA* American Society of Anesthesiologists physical status classification system, *E.N.T* Otolaryngology head, and neck surgery, *SBP* Systolic blood pressure, *MAP* Mean arterial pressure, *WBC* White blood cell, *ESR* Erythrocyte sedimentation rate, *Glu* Glucose, *Cre* Creatinine, *AST* Aspartate aminotransferase, *ALT* Alanine aminotransferase, *NSAIDs* Non-steroidal anti-inflammatory drugs

### Correlation between the SII and POD

To investigate the correlation between the SII and POD, we performed four logistic regression models. As a continuous variable, the SII was associated with POD in the unadjusted regression model with an odds ratio (OR) of 1.0004 (95 CI: 1.0003–1.0004), *P* < 0.001. In the multivariate logistic regression models, the ORs of SII were 1.0001 (model 1), 1.0002 (model 2), and 1.0001(model 3). So, the SII was an independent risk factor of POD (All *P* < 0.01, Additional file Table. A1).

The SII was divided into four categories using quartiles (SII ≤ 289, 289 < SII ≤ 416, 416 < SII ≤ 630, SII > 630). An elevated SII value was also associated with an increased risk of POD in all models. In the univariate analysis, the patients in the 416 < SII ≤ 630 and SII > 630 groups had a significantly higher risk of POD than those in the SII ≤ 289 group (P < 0.05). After adjusting for a series of variables (model 3), SII > 630 group still showed a significantly higher risk than SII ≤ 289(OR = 1.26, *P* = 0.038). All the detailed results are shown in Table A2 in the additional file.

The ROC of SII showed the optimal cut-off value for predicting POD is 650 (Figure A1 in the additional file). So, the SII was divided into two groups with a cut-off value of 650. The OR of SII > 650 group was 2.71 (95% CI: 2.37–3.09, *P* < 0.001) in the univariate analysis. In the multivariate logistic regression models, the OR of an SII value > 650 ranged from 1.32 to 1.86, and the *P* values were ≤ 0.001 for all models (Table 2). The overall univariate and multivariate logistic regression results are shown in Table A3 of additional file.

**Table 2:** Association between SII and POD with logistic regression models and PSM analysis

**Model**	**OR**^a^	**95%CI**	***P***
**Unadjusted model**	2.709	2.373–3.092	< 0.001
**Model 1 (adjusted for preoperative variables)**^b^	1.615	1.384–1.882	< 0.001
**Model 2 (adjusted for intraoperative variables)**^c^	1.855	1.602–2.146	< 0.001
**Model 3 (adjusted all the variables)**^d^	1.302	1.106–1.531	0.001
**Model PSM (*n* = 10,062)**^e^	1.301	1.062–1.598	0.011

*SII* Systemic-immune-inflammation index, *POD* Postoperative delirium, *OR* Odds ratio, *CI* Confidence interval, *PSM* Propensity score matching

^a^The ORs of SII > 650

^b^Model 1 adjusted for age, sex, BMI, ASA, alcohol, cerebrovascular disease, COPD, depression and anxiety, antipsychotic drugs, benzodiazepines, hemoglobin, WBC count, albumin, AST, ALT, PT, types of surgery, and anesthesia methods

^c^Model 2 adjusted for emergency surgery, duration of anesthesia, colloid, blood transfusion, duration of SBP > 140 mmHg, and MAP < 60 mmHg

^d^Model 3 adjusted for model 1 plus model 2

^e^Model PSM was a univariate regression model

### Propensity score matching analysis and adjustment

**Fig. 2:**
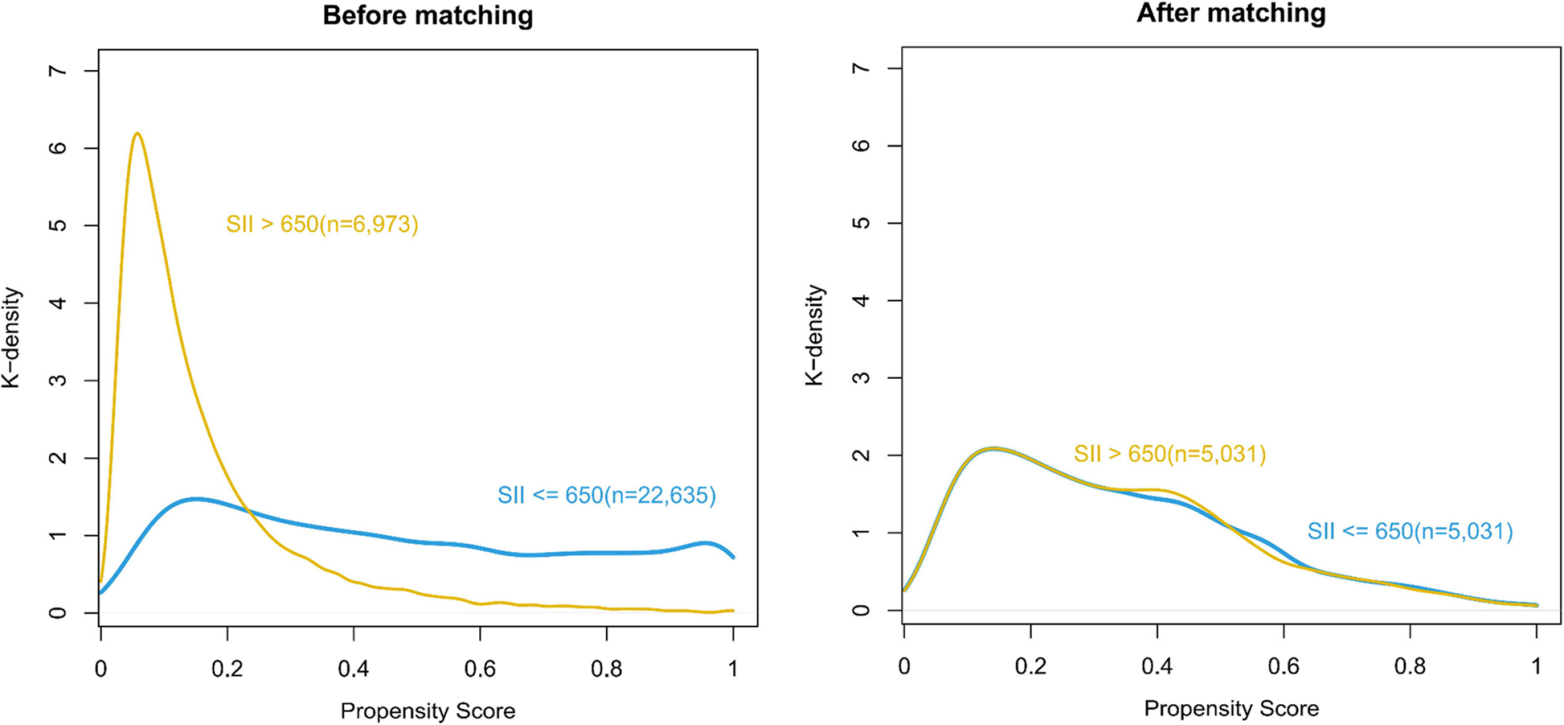
Distribution of propensity scores before and after matching. SII, systemic-immune-inflammation

Then, PSM analysis was performed as planned. The 14 variables (sex, age, albumin, emergency, type of surgery, WBC, Glu, Hb, BMI, total bilirubin, non-independent functional status, diabetes, type of surgery, duration of anesthesia) were matched in constructing PSM cohort. A total of 5031 patients in the SII ≤ 650 group were matched with 5031 patients in the SII > 650 group. The distribution of propensity scores of the patients before and after PSM is displayed in Fig. 2. The baseline characteristics and variables were balanced between the two groups (SMD < 0.1 except for the type of surgery) (Table 1). In the logistic regression performed after PSM, the SII was still an independent predictor of POD, with an OR of 1.301 (95% CI: 1.062–1.598, *P* = 0.011) (Table 2). The univariate logistic regression results are shown in Table A4 of additional file.

### Subgroup analyses

**Fig. 3:**
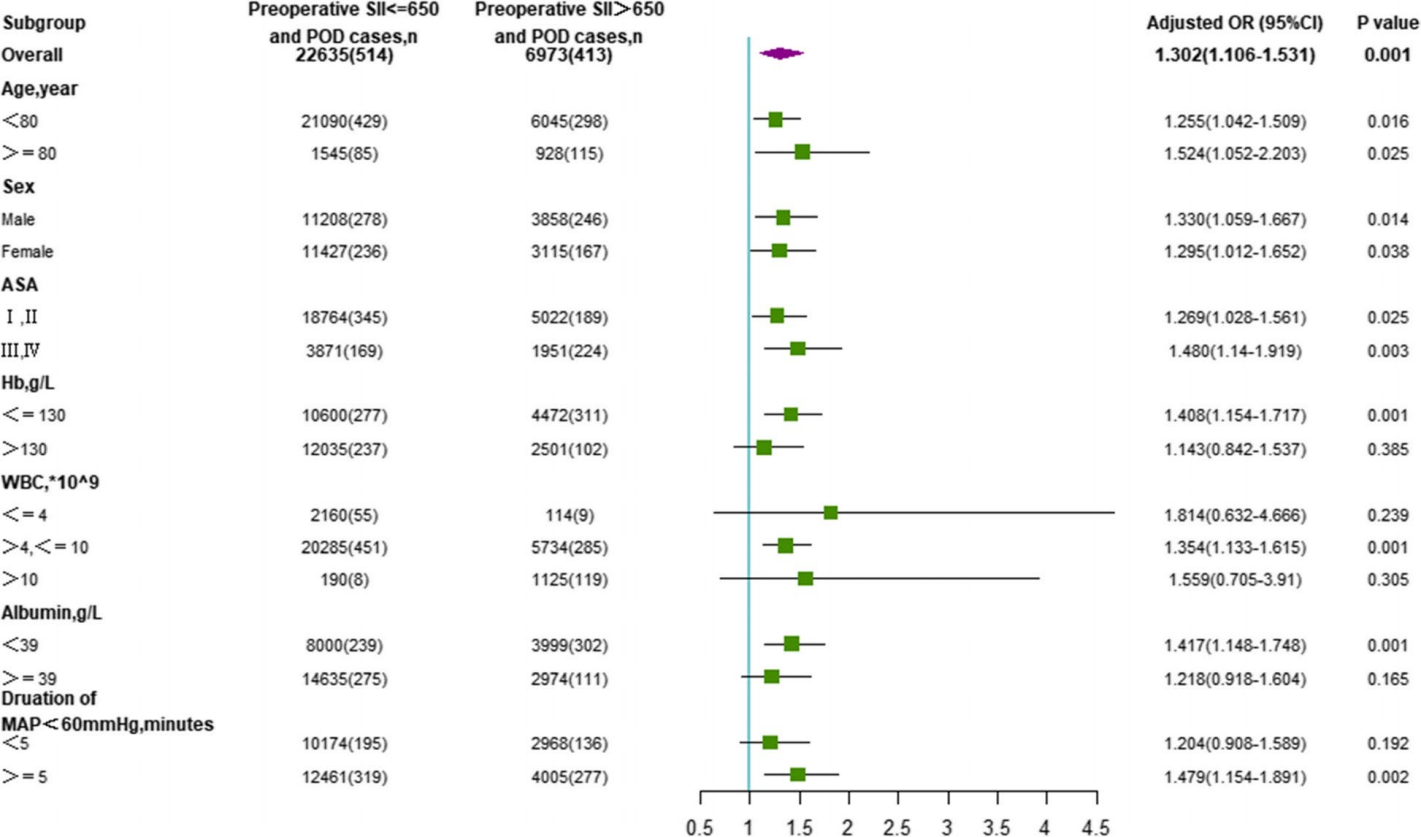
Subgroup analyses of the association between SII and perioperative ischemic stroke. SII, systemic-immune-inflammation; POD, postoperative delirium; OR, odds ratio; CI, Confidence interval; ASA, American Society of Anesthesiologists classification; Hb, hemoglobin; WBC, white blood cell count; MAP, mean arterial pressure

Additional subgroup analyses showed that the OR of the SII was significant for age, sex, and ASA classification in each subgroup (all *P* < 0.05, Fig. 3). The crude ORs of the SII generated in each subgroup showed that the associations between the SII and POD were stronger when albumin was < 39 g/L, the duration of a MAP < 60 mmHg was > 5 min, and hemoglobin was < 130 g/L (Fig. 3). In the three subgroups of WBC count, the OR of the SII was significant only in the 4 < WBC < 10 group (Fig. 3).

## Discussion

We evaluated the association between the SII and POD in 29,608 elderly patients who underwent nonneurosurgery and non-cardiac surgery with anaesthesia within our retrospective database. We found that the SII was an excellent predictor of POD after multivariate logistic regression and PSM.

The ability of SII as a continuous variable in predicting POD showed statistically significant in the univariate and 3 different multivariate models(*P* < 0.001). When the SII was divided into four categories with quartiles, it had an increasing trend of POD incidence as the SII grew in all models(P < 0.05). Then we explored the relationship between POD and SII as a categorical variable with a cut-off value of 650. In the univariate logistic regression, the SII > 650 showed a significant prediction for POD (OR 2.709, 95% CI 2.373–3.092, *P* < 0.001). Next, the association between the SII and POD was evaluated in 3 multivariate logistic regression models, including preoperative, intraoperative, and all variables. The results showed that the SII > 650 was still an independent risk factor for POD in elderly patients (*P* ≤ 0.001). To eliminate the confounding effect of covariates, we performed the PSM analysis further to test the relationship between the SII and POD. After all the covariates were used to estimate the propensity score, 5031 pairs of patients were matched. The results showed that the SII could be a powerful predictor of POD in the new cohort. The subgroup analysis indicated that an SII > 650 was associated with a significantly increased risk of POD in patients with an Hb level < 130 g/L, albumin level < 39 g/L, or a duration of MAP < 60 mmHg ≥ 5 min. In the 4 < WBC ≤ 10 group, patients with an SII ≥ 650 had an increased risk of POD (*P* < 0.001). The SII was found to be a valuable prognostic predictor for POD in patients of different ages, sexes, and ASA classifications.

Several possible mechanisms may account for the association between the preoperative SII and POD. First, Increased neutrophil levels, decreased lymphocyte levels, and decreased platelet levels are often nonspecific response to injury by trauma, surgery, and anesthesia [22, 23]. This change in peripheral circulation may involve disrupting the blood–brain-barrier and microglia, which activates and releases cerebral cytokines, resulting in POD [22, 24]. Second, many studies have found that white-cell derived inflammatory markers, such as the NLR, PLR, and PWR, are associated with delirium [11–15]. As one of the white-cell derived inflammatory markers, the SII was also calculated using neutrophil, lymphocyte, and platelet counts. Therefore, an elevated SII value indicated more severe immune-mediated and inflammation-mediated brain damage, which plays a critical role in the pathogenesis of POD [5, 22]. Moreover, many studies have indicated that the SII is associated with different cancers [25–27], ischaemic stroke [18, 28], and cerebral small vessel disease [16, 29, 30]. Individuals suffering from small vessel disease have more fragile brains, in which areas of vascular dysfunction can be more susceptible to acute impairment following systemic inflammatory insults [31, 32]. Neuroinflammation in areas of existing brain disease contributes to delirium [33]. As mentioned above, the SII may have good predictive power for POD.

To our knowledge, no study has explored the association between the SII and POD. Our analysis gave strong evidence to support that the SII can be a useful predictor of POD. As the incidence of POD differed among surgeries, we assessed the predictive ability of the SII for POD in an extensive retrospective cohort of 29,608 non-neurosurgery and non-cardiac surgery patients. Many statistical methods were used to confirm the conclusion. In addition to the multivariable logistic regression method, we also used PSM and subgroup analysis to reconfirm the conclusion. All the evidence proved that the SII was an independent predictor of POD in elderly patients undergoing non-neurosurgery and non-cardiac surgery.

However, our study also had some limitations. First, it is a retrospective study. The patients with POD were identified based on medical and nursing records but not assessment tools. The nurses may miss delirium for a lack of knowledge of POD assessment [34]. A gap existed between physicians’ perceptions and performance of delirium assessments in China. The actual prevalence of delirium assessment was suboptimal [35]. Before the evaluation of delirium becomes a routine postoperative evaluation item, only a retrospective dataset could provide such a large sample size. Nevertheless, only the recorded hyperactive and mixed POD patients needed urgent intervention. Therefore, predicting POD in these patients with the SII is also meaningful. We currently conduct a multicentre prospective study identifying POD patients with the Confusion Assessment Method (CAM) and 3D-CAM. Second, we only found an association between the SII and POD after a series of statistical analyses. The mechanism and validation of the association remain to be further studied. Thirdly, we only collected part of preoperative drugs and didn’t consider the effects of polypharmacy. Polypharmacy is also associated with delirium incidence and persistence [36]. In the future, we will try to collect all the preoperative drugs and fully consider polypharmacy.

## Conclusion

The SII was found to have a predictive value for POD in patients undergoing non-neurosurgery and non-cardiac surgery. As an index generated from routine blood tests, the SII has advantages in terms of cost and time required. After further validation, the SII may provide a new option for POD prediction.

## Supplementary Information

**Additional file 1:** **Figure A1.** The receiver operating characteristics (ROC) curve of SII for POD. **Table A1.** Association between SII as continuous variable and POD in different models. **Table A2.** Association between SII as categories variables used quartiles and POD in different models. **Table A3.** Association between SII as categories variables with a cut-off value of 650 and POD in different models. **Table A4.** Univariate logistic regression analyses for POD in the Model PSM.

## Data Availability

The datasets generated and/or analysed during the current study are not publicly available as individual privacy could be compromised but are available from the corresponding author on reasonable request.

## Abbreviations

OR: Odds ratio
CI: Confidence interval
SII: Systemic-immune-inflammation index
SMD: Standardized mean difference
PSM: Propensity Score Matching
BMI: Body mass index
COPD: Chronic obstructive pulmonary disease
CKD: Chronic kidney disease
ASA: American Society of Anaesthesiologists classification system
E.N.T: Otolaryngology head, and neck surgery
SBP: Systolic blood pressure
MAP: Mean arterial pressure
WBC: White blood cell
ESR: Erythrocyte sedimentation rate
Glu: Glucose
Cre: Creatinine
AST: Aspartate aminotransferase
ALT: Alanine aminotransferase
NSAIDs: Non-steroidal anti-inflammatory drugs
CAM: Confusion assessment method
