# Supplementary material for: Systemic immune-inflammation index predicts postoperative delirium in elderly patients after surgery: a retrospective cohort study
Source: BMC Geriatr. 2022 Sep 5;22:730. doi: 10.1186/s12877-022-03418-4 (PMC9446812; doi:10.1186/s12877-022-03418-4)
Supplement: Supplementary file 1 — Additional file 1: Figure A1. The receiver operating characteristics (ROC) curve of SII for POD. Table A1. Association between SII as continuous variable and POD in different models. Table A2. Association between SII as categories variables used quartiles and POD in different models. Table A3. Association between SII as categories variables with a cut-off value of 650 and POD in different models. Table A4. Univariate logistic regression analyses for POD in the Model PSM. [file 12877_2022_3418_MOESM1_ESM.docx]

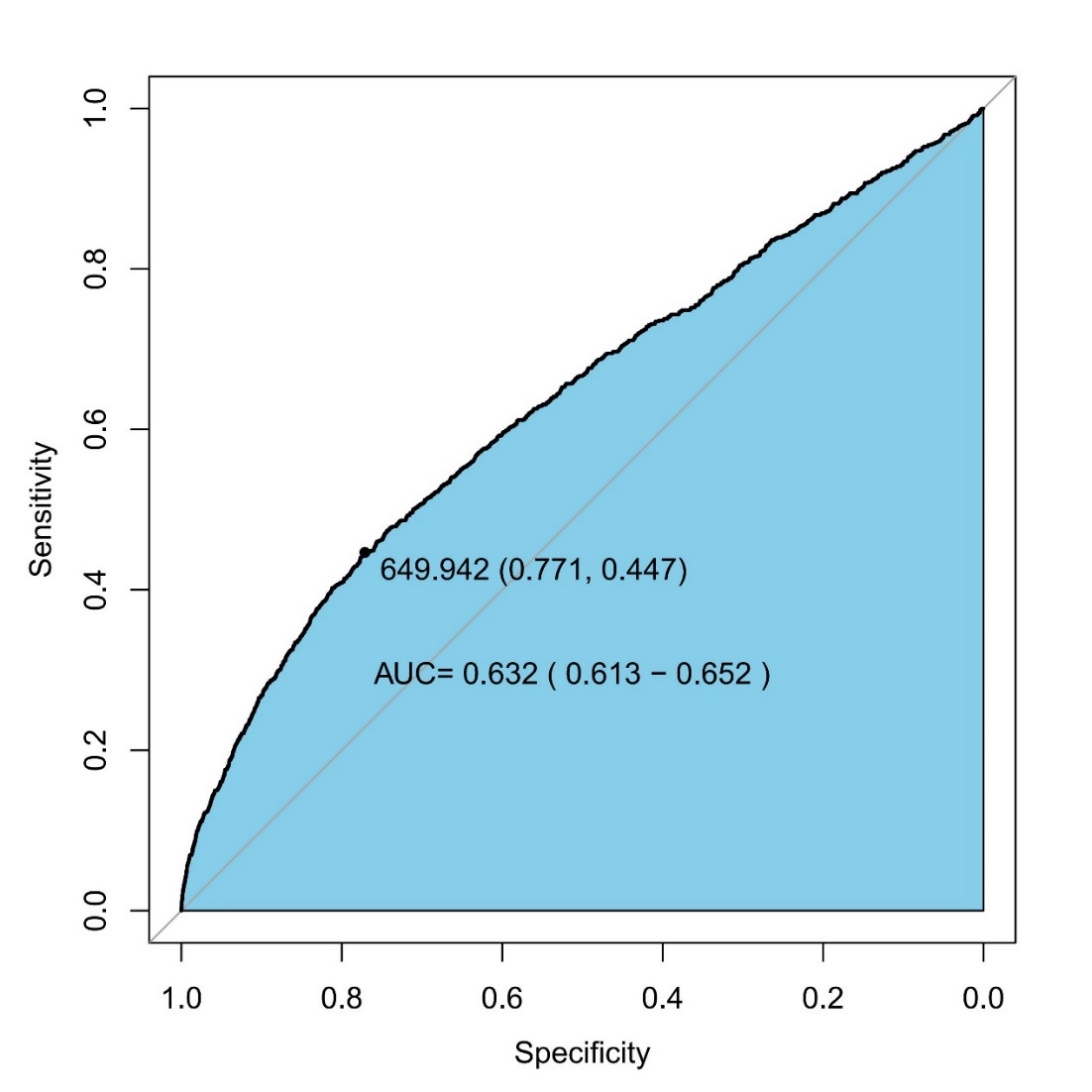


**Figure A1 The receiver operating characteristics (ROC) curve of SII for POD.**

The ROC curve showing the performance of the SII in predicting POD. Cutoff value= 649.9

SII, systemic-immune-inflammation index; POD, postoperative delirium; AUC, area under curve.

**Table A1** **Association between SII as continuous variable and POD in different models.**

| **Variables** | **Univariate analysis** | | **Model 1** | | **Model 2** | | **Model 3** | |
| --- | --- | --- | --- | --- | --- | --- | --- | --- |
|  | **OR (95%CI)** | ***P*** | **OR (95%CI)** | ***P*** | **OR (95%CI)** | ***P*** | **OR (95%CI)** | ***P*** |
| **SII** | 1.0004(1.0003-1.0004) | <0.001 | 1.000(1.000-1.000) | <0.001 | 1.000(1.000-1.000) | <0.001 | 1.00007(1.00001-1.000130) | 0.009 |
| **Age** | 1.089(1.078-1.1) | <0.001 | 1.049(1.036-1.062) | <0.001 |  |  | 1.062(1.048-1.075) | 1.062 |
| **Male (vs female)** | 1.264(1.108-1.443) | <0.001 | 1.219(1.022-1.455) | 0.028 |  |  | 1.082 (0.908-1.290) | 1.082 |
| **BMI** | 0.942(0.924-0.96) | <0.001 | 0.993(0.973-1.013) | 0.504 |  |  | 0.988 (0.968-1.008) | 0.240 |
| **Smoke (yes vs no)** | 1.238(1.067-1.432) | 0.004 | 1.16(0.954-1.409) | 0.134 |  |  | 1.115 (0.931-1.332) | 0.234 |
| **Alcohol (yes vs no)** | 1.161(0.995-1.35) | 0.055 | 1.06(0.868-1.291) | 0.567 |  |  |  |  |
| **Hypertension (yes vs no)** | 1.181(1.036-1.346) | 0.013 | 1.063(0.916-1.233) | 0.421 |  |  | 1.077(0.926-1.252) | 0.335 |
| **Diabetes (yes vs no)** | 1.226(1.057-1.418) | 0.007 | 0.983(0.827-1.163) | 0.839 |  |  | 1.023(0.861-1.213) | 0.792 |
| **Cardiovascular diseases (yes vs no)** | 1.34(1.093-1.627) | 0.004 | 0.981(0.786-1.214) | 0.865 |  |  | 0.966 (0.770-1.201) | 0.761 |
| **COPD (yes vs no)** | 2.531(1.996-3.17) | <0.001 | 2.462(1.928-3.107) | <0.001 |  |  | 1.894 (1.453-2.440) | <0.001 |
| **Cerebrovascular disease (yes vs no)** | 1.629(1.348-1.954) | <0.001 | 1.316(1.072-1.604) | 0.008 |  |  | 1.385 (1.125-1.694) | 0.002 |
| **CKD (yes vs no)** | 3.072(2.085-4.371) | <0.001 | 1.391(0.841-2.211) | 0.180 |  |  | 1.253 (0.749-2.013) | 0.370 |
| **Depression and anxiety (yes vs no)** | 2.976(1.638-4.983) | <0.001 | 1.528(0.723-2.929) | 0.233 |  |  | 1.556 (0.7183-3.046) | 0.228 |
| **Non-independent functional status (yes vs no)** | 1.97(1.726-2.247) | <0.001 | 1.151(0.987-1.342) | 0.073 |  |  | 1.146(0.980-1.339) | 0.086 |
| **ASA (vs I)** |  |  |  |  |  |  |  |  |
| **II** | 1.151(0.586-2.703) | 0.715 | 0.919(0.465-2.17) | 0.828 |  |  | 0.767 (0.386-1.813) | 0.494 |
| **III** | 3.25(1.649-7.655) | 0.002 | 1.518(0.76-3.607) | 0.286 |  |  | 1.163 (0.580-2.768) | 0.701 |
| **IV** | 15.179(7.192-37.315) | <0.001 | 4.145(1.899-10.425) | 0.001 |  |  | 2.685 (1.209-6.831) | 0.023 |
| **Days in hospital before surgery** | 1.003(1-1.008) | 0.043 | 1.002(0.993-1.006) | 0.385 |  |  | 1.000(0.989-1.005) | 0.934 |
| **Emergency surgery, (yes vs no)** | 4.341(3.437-5.423) | <0.001 |  |  | 3.809(2.905-4.939) | <0.001 | 2.518(1.838-3.409) | <0.001 |
| **Hemoglobin** | 0.976(0.973-0.98) | <0.001 | 0.993(0.989-0.998) | 0.004 |  |  | 0.995 (0.990-0.999) | 0.038 |
| **WBC count** | 1.13(1.106-1.154) | <0.001 | 1.052(1.032-1.074) | <0.001 |  |  | 1.043 (1.024-1.063) | <0.001 |
| **Monocytes** | 0.302(0.01-2.334) | 0.48 | 1.441(0.254-3.11) | 0.435 |  |  |  |  |
| **Glu** | 1.141(1.111-1.17) | <0.001 | 1.083(1.048-1.118) | <0.001 |  |  | 1.059 (1.022-1.095) | 0.001 |
| **Albumin** | 0.881(0.867-0.894) | <0.001 | 0.95(0.932-0.969) | <0.001 |  |  | 0.965 (0.946-0.984) | <0.001 |
| **Cre** | 1.004(1.002-1.005) | <0.001 | 1(0.998-1.002) | 0.796 |  |  | 1.001 (0.999-1.003) | 0.382 |
| **Total bilirubin** | 1.003(1.002-1.005) | <0.001 | 1.002(1-1.003) | 0.087 |  |  | 1.000 (0.998-1.002) | 0.975 |
| **AST** | 1.001(0.999-1.002) | 0.344 | 0.994(0.99-0.998) | 0.003 |  |  |  | 0.707 |
| **ALT** | 1.003(1.001-1.004) | <0.001 | 1.005(1.001-1.008) | 0.007 |  |  | 1.000 (0.997-1.002) | 0.108 |
| **PT** | 1.2(1.15-1.253) | <0.001 | 1.05(0.997-1.099) | 0.048 |  |  | 1.044 (0.987-1.095) | 0.001 |
| **Preoperative medication** |  |  |  |  |  |  |  |  |
| **Anticholinergic drug (yes vs no)** | 0.914(0.802-1.043) | 0.18 | 0.931(0.811-1.07) | 0.314 |  |  |  |  |
| **NSAIDs (yes vs no)** | 0.725(0.532-0.964) | 0.034 | 0.807(0.573-1.113) | 0.206 |  |  |  |  |
| **Benzodiazepines (yes vs no)** | 1.154(0.99-1.34) | 0.064 | 1.222(1.019-1.46) | 0.029 |  |  | 0.882 (0.625-1.219) |  |
| **Antipsychotic drugs (yes vs no)** | 72.022(48.077-110.042) | <0.001 | 48.679(31.138-77.404) | <0.001 |  |  | 46.940 (29.738-75.417) | <0.001 |
| **Type of surgery (vs Hepatopancreatobiliary and gastrointestinal surgery)** |  |  |  |  |  |  |  |  |
| **Orthopedic surgery** | 0.804(0.687-0.939) | 0.006 | 0.992(0.801-1.225) | 0.941 |  |  | 1.002 (0.802-1.251) | 0.985 |
| **Urinary surgery** | 0.595(0.452-0.772) | <0.001 | 0.777(0.58-1.026) | 0.083 |  |  | 1.039 (0.770-1.384) | 0.797 |
| **Thoracic surgery** | 0.546(0.397-0.733) | <0.001 | 0.771(0.538-1.087) | 0.147 |  |  | 1.034 (0.730-1.434) | 0.847 |
| **E.N.T** | 0.349(0.218-0.529) | <0.001 | 0.432(0.264-0.67) | 0.000 |  |  | 0.669 (0.401-1.057) | 0.101 |
| **Gynecology** | 0.596(0.382-0.884) | 0.015 | 0.804(0.496-1.248) | 0.353 |  |  | 1.221 (0.752-1.897) | 0.395 |
| **Vascular surgery** | 1.029(0.749-1.382) | 0.855 | 1.103(0.775-1.536) | 0.575 |  |  | 1.367 (0.945-1.942) | 0.089 |
| **Others** | 0.481(0.349-0.648) | <0.001 | 0.681(0.488-0.931) | 0.020 |  |  | 1.094 (0.766-1.532) | 0.609 |
| **Anesthesia method (vs Basal anesthesia)** |  |  |  |  |  |  |  |  |
| **Epidural anesthesia** | 0.458(0.152-1.281) | 0.141 | 0.509(0.155-1.541) | 0.242 |  |  |  |  |
| **Nerve blocks** | 1.776(0.853-4.051) | 0.143 | 1.585(0.713-3.819) | 0.277 |  |  |  |  |
| **General anesthesia** | 1.331(0.728-2.797) | 0.399 | 2.381(1.249-5.169) | 0.016 |  |  |  |  |
| **General anesthesia combined with other anesthesia** | 1.314(0.699-2.812) | 0.436 | 1.618(0.819-3.598) | 0.198 |  |  |  |  |
| **Duration of surgery** | 1.004(1.004-1.005) | <0.001 |  |  | 1.000(0.998-1.002) | 0.807 | 1.001 (0.999-1.003) | 0.089 |
| **Duration of anesthesia** | 1.004(1.004-1.005) | <0.001 |  |  | 1.002(1.001-1.005) | 0.014 | 1.002 (1.000-1.003) | 0.012 |
| **Blood loss** | 1.001(1-1.001) | <0.001 |  |  | 1.000(1.000-1.000) | 0.523 | 1.000 (0.999-1.000) | 0.418 |
| **Urine** | 1.001(1-1.001) | <0.001 |  |  | 1.000(1.000-1.000) | 0.733 | 1.000 (0.999-1.000) | 0.615 |
| **Crystalloid** | 1(1-1.001) | <0.001 |  |  | 1.000(1.000-1.000) | 0.791 | 1.000(0.999-1.000) | 0.568 |
| **Colloid** | 1.001(1.001-1.001) | <0.001 |  |  | 1.000(1.000-1.001) | 0.001 | 1.000 (1.000-1.001) | <0.001 |
| **Blood transfusion** | 2.558(2.208-2.957) | <0.001 |  |  | 1.73(1.453-2.053) | <0.001 | 1.318(1.085-1.596) | 0.005 |
| **Duration of SBP>140 mmHg** | 1.007(1.006-1.009) | <0.001 |  |  | 1.004(1.002-1.006) | <0.001 | 1.001 (0.999-1.003) | 0.158 |
| **Duration of MAP<60 mmHg** | 1.016(1.013-1.019) | <0.001 |  |  | 1.007(1.004-1.01) | <0.001 | 1.005 (1.002-1.009) | 0.003 |
| **Intraoperative medication** |  |  |  |  |  |  |  |  |
| **Glucocorticoid** | 1.064(0.929-1.22) | 0.376 |  |  | 0.952(0.826-1.098) | 0.497 |  |  |
| **Dexmedetomidine** | 1.094(0.881-1.344) | 0.403 |  |  | 1.053(0.844-1.302) | 0.637 |  |  |

*Abbreviations:* SII, systemic-immune-inflammation index; OR, odds ratio; CI, confidence interval; BMI, body mass index; COPD, chronic obstructive pulmonary disease; CKD, chronic kidney disease; ASA, American Society of Anesthesiologists physical status classification system; E.N.T, otolaryngology head and neck surgery; SBP, systolic blood pressure; MAP, mean arterial pressure; WBC, white blood cell; ESR, erythrocyte sedimentation rate; Glu, glucose; Cre, creatinine; AST, aspartate aminotransferase; ALT, alanine aminotransferase; NSAIDs, non-steroidal anti-inflammatory drugs

**Table A2** **Association between SII as categories variables used quartiles and POD in different models.**

| **Variables** | **Univariate analysis** | | **Model 1** | | **Model 2** | | **Model 3** | |
| --- | --- | --- | --- | --- | --- | --- | --- | --- |
|  | **OR (95%CI)** | ***P*** | **OR (95%CI)** | ***P*** | **OR (95%CI)** | ***P*** | **OR (95%CI)** | ***P*** |
| **SII(vs** **SII≤289)** |  |  |  |  |  |  |  |  |
| **289<SII≤416** | 1.082(0.864-1.356) | 0.493 | 1.022(0.81-1.288) | 0.857 | 1.035(0.825-1.3) | 0.766 | 0.974(0.771-1.232) | 0.828 |
| **416<SII≤630** | 1.301(1.049-1.617) | 0.017 | 1.098(0.879-1.374) | 0.410 | 1.115(0.896-1.39) | 0.330 | 0.994(0.793-1.248) | 0.960 |
| **SII>630** | 2.963(2.458-3.591) | <0.001 | 1.632(1.329-2.013) | <0.001 | 1.904(1.557-2.337) | <0.001 | 1.261(1.014-1.572) | 0.038 |
| **Age** | 1.089(1.078-1.1) | <0.001 | 1.047(1.034-1.060) | <0.001 |  |  | 1.06(1.047-1.074) | <0.001 |
| **Male (vs female)** | 1.264(1.108-1.443) | <0.001 | 1.191(0.998-1.422) | 0.052 |  |  | 1.076(0.898-1.29) | 0.428 |
| **BMI** | 0.942(0.924-0.96) | <0.001 | 0.993(0.973-1.013) | 0.473 |  |  | 1.176(0.585-2.807) | 0.681 |
| **Smoke (yes vs no)** | 1.238(1.067-1.432) | 0.004 | 1.164(0.958-1.412) | 0.125 |  |  | 2.692(1.21-6.862) | 0.023 |
| **Alcohol (yes vs no)** | 1.161(0.995-1.35) | 0.055 | 1.038(0.85-1.264) | 0.713 |  |  | 1.162(0.992-1.361) | 0.063 |
| **Hypertension (yes vs no)** | 1.181(1.036-1.346) | 0.013 | 1.061(0.915-1.231) | 0.430 |  |  | 0.988(0.967-1.008) | 0.237 |
| **Diabetes (yes vs no)** | 1.226(1.057-1.418) | 0.007 | 0.986(0.831-1.167) | 0.871 |  |  | 1.103(0.903-1.345) | 0.333 |
| **Cardiovascular diseases (yes vs no)** | 1.34(1.093-1.627) | 0.004 | 0.983(0.788-1.216) | 0.878 |  |  | 1.017(0.83-1.244) | 0.868 |
| **COPD (yes vs no)** | 2.531(1.996-3.17) | <0.001 | 2.376(1.86-2.997) | <0.001 |  |  | 1.379(1.12-1.687) | 0.002 |
| **Cerebrovascular disease (yes vs no)** | 1.629(1.348-1.954) | <0.001 | 1.298(1.057-1.582) | 0.011 |  |  | 1.075(0.924-1.25) | 0.349 |
| **CKD (yes vs no)** | 3.072(2.085-4.371) | <0.001 | 1.395(0.844-2.213) | 0.175 |  |  | 0.974(0.777-1.212) | 0.819 |
| **Depression and anxiety (yes vs no)** | 2.976(1.638-4.983) | <0.001 | 1.563(0.743-2.984) | 0.206 |  |  | 1.034(0.869-1.227) | 0.702 |
| **Non-independent functional status (yes vs no)** | 1.97(1.726-2.247) | <0.001 | 1.12(0.96-1.306) | 0.149 |  |  | 0.779(0.391-1.846) | 0.521 |
| **ASA (vs I)** |  |  |  |  |  |  |  |  |
| **II** | 1.151(0.586-2.703) | 0.715 | 0.906(0.458-2.14) | 0.799 |  |  | 1.035(0.723-1.451) | 0.848 |
| **III** | 3.25(1.649-7.655) | 0.002 | 1.502(0.751-3.569) | 0.299 |  |  | 47.775(30.305-76.665) | <0.001 |
| **IV** | 15.179(7.192-37.315) | <0.001 | 4.085(1.875-10.261) | 0.001 |  |  | 1.892(1.451-2.439) | <0.001 |
| **Days in hospital before surgery** | 1.003(1-1.008) | 0.043 | 1.002(0.993-1.006) | 0.383 |  |  | 1.002(1-1.004) | 0.066 |
| **Emergency surgery, (yes vs no)** | 4.341(3.437-5.423) | <0.001 |  |  | 3.913(3.018-5.025) | <0.001 | 0.469(0.139-1.45) | 0.197 |
| **Hemoglobin** | 0.976(0.973-0.98) | <0.001 | 0.995(0.99-0.999) | 0.023 |  |  | 0.866(0.612-1.199) | 0.401 |
| **WBC count** | 1.13(1.106-1.154) | <0.001 | 1.051(1.032-1.072) | <0.001 |  |  | 0.996(0.991-1.001) | 0.099 |
| **Monocytes** | 0.302(0.01-2.334) | 0.480 | 1.268(0.171-2.788) | 0.652 |  |  | 1.061(1.024-1.098) | 0.001 |
| **Glu** | 1.141(1.111-1.17) | <0.001 | 1.082(1.046-1.117) | <0.001 |  |  | 0.963(0.944-0.982) | 0.000 |
| **Albumin** | 0.881(0.867-0.894) | <0.001 | 0.95(0.932-0.969) | <0.001 |  |  | 1.001(0.999-1.003) | 0.386 |
| **Cre** | 1.004(1.002-1.005) | <0.001 | 1.000(0.998-1.002) | 0.863 |  |  | 1.001(0.999-1.003) | 0.405 |
| **Total bilirubin** | 1.003(1.002-1.005) | <0.001 | 1.001(0.999-1.003) | 0.213 |  |  | 0.994(0.99-0.998) | 0.004 |
| **AST** | 1.001(0.999-1.002) | 0.344 | 0.994(0.99-0.998) | 0.003 |  |  | 1.004(1.001-1.008) | 0.016 |
| **ALT** | 1.003(1.001-1.004) | <0.001 | 1.005(1.001-1.008) | 0.006 |  |  | 1.046(0.989-1.098) | 0.091 |
| **PT** | 1.200(1.15-1.253) | <0.001 | 1.057(1.005-1.105) | 0.020 |  |  | 1.000(0.987-1.005) | 0.979 |
| **Preoperative medication** |  |  |  |  |  |  |  |  |
| **Anticholinergic drug (yes vs no)** | 0.914(0.802-1.043) | 0.180 | 0.96(0.836-1.102) | 0.560 |  |  | 1.68(0.786-3.248) | 0.150 |
| **NSAIDs (yes vs no)** | 0.725(0.532-0.964) | 0.034 | 0.818(0.581-1.127) | 0.234 |  |  | 1.202(0.999-1.44) | 0.048 |
| **Benzodiazepines (yes vs no)** | 1.154(0.99-1.34) | 0.064 | 1.227(1.023-1.465) | 0.025 |  |  | 1.255(0.750-2.017) | 0.367 |
| **Antipsychotic drugs (yes vs no)** | 72.022(48.077-110.042) | <0.001 | 49.315(31.524-78.47) | <0.001 |  |  | 1.006(1.002-1.009) | 0.002 |
| **Type of surgery (vs Hepatopancreatobiliary and gastrointestinal surgery)** |  |  |  |  |  |  |  |  |
| **Orthopedic surgery** | 0.804(0.687-0.939) | 0.006 | 0.974(0.786-1.203) | 0.809 |  |  | 1.468(0.652-3.582) | 0.372 |
| **Urinary surgery** | 0.595(0.452-0.772) | <0.001 | 0.779(0.581-1.029) | 0.087 |  |  | 1.501(0.768-3.327) | 0.273 |
| **Thoracic surgery** | 0.546(0.397-0.733) | <0.001 | 0.756(0.527-1.065) | 0.118 |  |  | 1.369(0.682-3.088) | 0.411 |
| **E.N.T** | 0.349(0.218-0.529) | <0.001 | 0.443(0.271-0.686) | 0.001 |  |  | 1.009(0.79-1.285) | 0.943 |
| **Gynecology** | 0.596(0.382-0.884) | 0.015 | 0.805(0.497-1.25) | 0.356 |  |  | 1.043(0.771-1.39) | 0.780 |
| **Vascular surgery** | 1.029(0.749-1.382) | 0.855 | 1.119(0.787-1.558) | 0.519 |  |  | 0.810(0.555-1.164) | 0.264 |
| **Others** | 0.481(0.349-0.648) | <0.001 | 0.69(0.493-0.943) | 0.024 |  |  | 0.630(0.378-0.996) | 0.060 |
| **Anesthesia method (vs Basal anesthesia)** |  |  |  |  |  |  |  |  |
| **Epidural anesthesia** | 0.458(0.152-1.281) | 0.141 | 0.526(0.159-1.597) | 0.266 |  |  | 1.206(1.03-1.414) | 0.021 |
| **Nerve blocks** | 1.776(0.853-4.051) | 0.143 | 1.583(0.711-3.817) | 0.279 |  |  | 1.043(1.024-1.063) | <0.001 |
| **General anesthesia** | 1.331(0.728-2.797) | 0.399 | 2.461(1.289-5.345) | 0.012 |  |  | 0.645(0.021-2.504) | 0.777 |
| **General anesthesia combined with other anesthesia** | 1.314(0.699-2.812) | 0.436 | 1.627(0.823-3.623) | 0.193 |  |  | 2.587(1.891-3.5) | <0.001 |
| **Duration of surgery** | 1.004(1.004-1.005) | <0.001 |  |  | 1.000(0.998-1.002) | 0.933 | 1.021(0.62-1.61) | 0.933 |
| **Duration of anesthesia** | 1.004(1.004-1.005) | <0.001 |  |  | 1.002(1.001-1.005) | 0.013 | 1.429(0.978-2.05) | 0.058 |
| **Blood loss** | 1.001(1.000-1.001) | <0.001 |  |  | 1.000(1.000-1.000) | 0.531 | 1.002(1.000-1.003) | 0.016 |
| **Urine** | 1.001(1.000-1.001) | <0.001 |  |  | 1.000(1.000-1.000) | 0.559 | 1.001(0.999-1.003) | 0.100 |
| **Crystalloid** | 1.000(1.000-1.001) | <0.001 |  |  | 1.000(1.000-1.000) | 0.710 | 1.000(1.000-1.000) | 0.454 |
| **Colloid** | 1.001(1.001-1.001) | <0.001 |  |  | 1.000(1.000-1.001) | 0.001 | 1.000(1.000-1.000) | 0.837 |
| **Blood transfusion** | 2.558(2.208-2.957) | <0.001 |  |  | 1.685(1.417-1.997) | <0.001 | 1.000(1.000-1.001) | 0.000 |
| **Duration of SBP>140 mmHg** | 1.007(1.006-1.009) | <0.001 |  |  | 1.004(1.002-1.006) | <0.001 | 1.000(1.000-1.000) | 0.785 |
| **Duration of MAP<60 mmHg** | 1.016(1.013-1.019) | <0.001 |  |  | 1.007(1.003-1.010) | <0.001 | 1.291(1.063-1.564) | 0.010 |
| **Intraoperative medication** |  |  |  |  |  |  |  |  |
| **Glucocorticoid** | 1.064(0.929-1.22) | 0.376 |  |  | 0.965(0.837-1.113) | 0.619 | 1.010(0.866-1.180) | 0.899 |
| **Dexmedetomidine** | 1.094(0.881-1.344) | 0.403 |  |  | 1.044(0.836-1.291) | 0.694 | 1.074(0.848-1.346) | 0.546 |

*Abbreviations:* SII, systemic-immune-inflammation index; OR, odds ratio; CI, confidence interval; BMI, body mass index; COPD, chronic obstructive pulmonary disease; CKD, chronic kidney disease; ASA, American Society of Anesthesiologists physical status classification system; E.N.T, otolaryngology head and neck surgery; SBP, systolic blood pressure; MAP, mean arterial pressure; WBC, white blood cell; ESR, erythrocyte sedimentation rate; Glu, glucose; Cre, creatinine; AST, aspartate aminotransferase; ALT, alanine aminotransferase; NSAIDs, non-steroidal anti-inflammatory drugs

**Table A3** **Association between SII as categories variables with a cut-off value of 650 and POD in different models.**

| **Variables** | **Univariate analysis** | | **Model 1** | | **Model 2** | | **Model 3** | |
| --- | --- | --- | --- | --- | --- | --- | --- | --- |
|  | **OR (95%CI)** | ***P*** | **OR (95%CI)** | ***P*** | **OR (95%CI)** | ***P*** | **OR (95%CI)** | ***P*** |
| **SII (****≤650 vs >650)** | 2.709(2.373-3.092) | <0.001 | 1.615(1.384-1.882) | <0.001 | 1.855(1.602-2.146) | <0.001 | 1.302(1.106-1.531) | 0.001 |
| **Age** | 1.089(1.078-1.1) | <0.001 | 1.047(1.034-1.06) | <0.001 |  |  | 1.061(1.047-1.074) | <0.001 |
| **Male (vs female)** | 1.264(1.108-1.443) | <0.001 | 1.193(1-1.424) | 0.050 |  |  | 1.067(0.895-1.273) | 0.466 |
| **BMI** | 0.942(0.924-0.96) | <0.001 | 0.993(0.973-1.013) | 0.494 |  |  | 0.988(0.968-1.008) | 0.248 |
| **Smoke (yes vs no)** | 1.238(1.067-1.432) | 0.004 | 1.163(0.957-1.412) | 0.126 |  |  | 1.114(0.931-1.331) | 0.235 |
| **Alcohol (yes vs no)** | 1.161(0.995-1.35) | 0.055 | 1.04(0.852-1.267) | 0.697 |  |  |  |  |
| **Hypertension (yes vs no)** | 1.181(1.036-1.346) | 0.013 | 1.064(0.918-1.234) | 0.410 |  |  | 1.076(0.925-1.251) | 0.342 |
| **Diabetes (yes vs no)** | 1.226(1.057-1.418) | 0.007 | 0.987(0.832-1.168) | 0.878 |  |  | 1.028(0.865-1.219) | 0.752 |
| **Cardiovascular diseases (yes vs no)** | 1.34(1.093-1.627) | 0.004 | 0.982(0.787-1.215) | 0.869 |  |  | 0.967(0.771-1.202) | 0.766 |
| **COPD (yes vs no)** | 2.531(1.996-3.17) | <0.001 | 2.378(1.862-3) | <0.001 |  |  | 1.872(1.436-2.411) | <0.001 |
| **Cerebrovascular disease (yes vs no)** | 1.629(1.348-1.954) | <0.001 | 1.3(1.059-1.585) | 0.011 |  |  | 1.380(1.121-1.687) | 0.002 |
| **CKD (yes vs no)** | 3.072(2.085-4.371) | <0.001 | 1.388(0.84-2.203) | 0.181 |  |  | 1.253(0.748-2.012) | 0.371 |
| **Depression and anxiety (yes vs no)** | 2.976(1.638-4.983) | <0.001 | 1.549(0.736-2.96) | 0.215 |  |  | 1.574(0.729-3.068) | 0.214 |
| **Non-independent functional status (yes vs no)** | 1.97(1.726-2.247) | <0.001 | 1.118(0.958-1.304) | 0.156 |  |  | 1.128(0.965-1.319) | 0.129 |
| **ASA (vs I)** |  |  |  |  |  |  |  |  |
| **II** | 1.151(0.586-2.703) | 0.715 | 0.909(0.459-2.148) | 0.806 |  |  |  |  |
| **III** | 3.25(1.649-7.655) | 0.002 | 1.512(0.756-3.595) | 0.291 |  |  |  |  |
| **IV** | 15.179(7.192-37.315) | <0.001 | 4.076(1.871-10.24) | 0.001 |  |  |  |  |
| **Days in hospital before surgery** | 1.003(1-1.008) | 0.043 | 1.002(0.993-1.006) | 0.391 |  |  | 1.000(0.989-1.005) | 0.905 |
| **Emergency surgery, (yes vs no)** | 4.341(3.437-5.423) | <0.001 |  |  | 3.896(3.003-5.004) | <0.001 | 2.590(1.905-3.484) | <0.001 |
| **Hemoglobin** | 0.976(0.973-0.98) | <0.001 | 0.995(0.99-0.999) | 0.024 |  |  | 0.996(0.991-1.001) | 0.085 |
| **WBC count** | 1.13(1.106-1.154) | <0.001 | 1.051(1.032-1.071) | <0.001 |  |  | 1.043(1.025-1.063) | <0.001 |
| **Monocytes** | 0.302(0.01-2.334) | 0.48 | 1.265(0.173-2.78) | 0.652 |  |  |  |  |
| **Glu** | 1.141(1.111-1.17) | <0.001 | 1.081(1.045-1.116) | <0.001 |  |  | 1.056(1.019-1.092) | 0.002 |
| **Albumin** | 0.881(0.867-0.894) | <0.001 | 0.951(0.932-0.969) | <0.001 |  |  | 0.966(0.947-0.985) | <0.001 |
| **Cre** | 1.004(1.002-1.005) | <0.001 | 1(0.998-1.002) | 0.852 |  |  | 1.001(0.999-1.003) | 0.403 |
| **Total bilirubin** | 1.003(1.002-1.005) | <0.001 | 1.001(0.999-1.003) | 0.216 |  |  | 0.999(0.998-1.002) | 0.849 |
| **AST** | 1.001(0.999-1.002) | 0.344 | 0.994(0.99-0.998) | 0.003 |  |  |  |  |
| **ALT** | 1.003(1.001-1.004) | <0.001 | 1.005(1.001-1.008) | 0.006 |  |  | 0.999(0.997-1.002) | 0.737 |
| **PT** | 1.2(1.15-1.253) | <0.001 | 1.056(1.004-1.104) | 0.024 |  |  | 1.046(0.99-1.097) | 0.086 |
| **Preoperative medication** |  |  |  |  |  |  |  |  |
| **Anticholinergic drug (yes vs no)** | 0.914(0.802-1.043) | 0.18 | 0.961(0.837-1.104) | 0.575 |  |  |  |  |
| **NSAIDs (yes vs no)** | 0.725(0.532-0.964) | 0.034 | 0.819(0.581-1.129) | 0.237 |  |  | 0.886(0.627-1.223) | 0.474 |
| **Benzodiazepines (yes vs no)** | 1.154(0.99-1.34) | 0.064 | 1.23(1.026-1.468) | 0.024 |  |  |  |  |
| **Antipsychotic drugs (yes vs no)** | 72.022(48.077-110.042) | <0.001 | 49.975(31.967-79.458) | <0.001 |  |  | 47.479(30.071-76.309) | <0.001 |
| **Type of surgery (vs Hepatopancreatobiliary and gastrointestinal surgery)** |  |  |  |  |  |  |  |  |
| **Orthopedic surgery** | 0.804(0.687-0.939) | 0.006 | 0.974(0.786-1.203) | 0.810 |  |  | 0.989(0.791-1.235) | 0.922 |
| **Urinary surgery** | 0.595(0.452-0.772) | <0.001 | 0.779(0.581-1.029) | 0.086 |  |  | 1.037(0.768-1.382) | 0.807 |
| **Thoracic surgery** | 0.546(0.397-0.733) | <0.001 | 0.752(0.525-1.06) | 0.112 |  |  | 1.019(0.72-1.415) | 0.911 |
| **E.N.T** | 0.349(0.218-0.529) | <0.001 | 0.44(0.269-0.682) | 0.001 |  |  | 0.665(0.399-1.05) | 0.096 |
| **Gynecology** | 0.596(0.382-0.884) | 0.015 | 0.808(0.498-1.254) | 0.363 |  |  | 1.221(0.752-1.897) | 0.396 |
| **Vascular surgery** | 1.029(0.749-1.382) | 0.855 | 1.115(0.785-1.554) | 0.530 |  |  | 1.358(0.938-1.93) | 0.096 |
| **Others** | 0.481(0.349-0.648) | <0.001 | 0.689(0.493-0.942) | 0.024 |  |  | 1.092(0.764-1.528) | 0.618 |
| **Anesthesia method (vs Basal anesthesia)** |  |  |  |  |  |  |  |  |
| **Epidural anesthesia** | 0.458(0.152-1.281) | 0.141 | 0.526(0.159-1.599) | 0.267 |  |  |  |  |
| **Nerve blocks** | 1.776(0.853-4.051) | 0.143 | 1.598(0.718-3.854) | 0.269 |  |  |  |  |
| **General anesthesia** | 1.331(0.728-2.797) | 0.399 | 2.477(1.297-5.382) | 0.012 |  |  |  |  |
| **General anesthesia combined with other anesthesia** | 1.314(0.699-2.812) | 0.436 | 1.638(0.828-3.646) | 0.188 |  |  |  |  |
| **Duration of surgery** | 1.004(1.004-1.005) | <0.001 |  |  | 1.000(0.998-1.002) | 0.938 | 1.001(1-1.003) | 0.100 |
| **Duration of anesthesia** | 1.004(1.004-1.005) | <0.001 |  |  | 1.002(1.001-1.005) | 0.012 | 1.002(1-1.003) | 0.011 |
| **Blood loss** | 1.001(1-1.001) | <0.001 |  |  | 1.000(1.000-1.000) | 0.515 | 0.999(1-1) | 0.377 |
| **Urine** | 1.001(1-1.001) | <0.001 |  |  | 1.000(1.000-1.000) | 0.577 | 1.000(1-1) | 0.588 |
| **Crystalloid** | 1(1-1.001) | <0.001 |  |  | 1.000(1.000-1.000) | 0.737 | 1.000(1-1) | 0.582 |
| **Colloid** | 1.001(1.001-1.001) | <0.001 |  |  | 1.000(1.000-1.001) | 0.001 | 1.000(1-1.001) | <0.001 |
| **Blood transfusion** | 2.558(2.208-2.957) | <0.001 |  |  | 1.682(1.415-1.994) | <0.001 | 1.318(1.086-1.596) | 0.005 |
| **Duration of SBP>140 mmHg** | 1.007(1.006-1.009) | <0.001 |  |  | 1.004(1.002-1.006) | <0.001 | 1.001(0.999-1.003) | 0.171 |
| **Duration of MAP<60 mmHg** | 1.016(1.013-1.019) | <0.001 |  |  | 1.007(1.003-1.010) | <0.001 | 1.005(1.002-1.009) | 0.003 |
| **Intraoperative medication** |  |  |  |  |  |  |  |  |
| **Glucocorticoid** | 1.064(0.929-1.22) | 0.376 |  |  | 0.965(0.838-1.114) | 0.627 |  |  |
| **Dexmedetomidine** | 1.094(0.881-1.344) | 0.403 |  |  | 1.048(0.839-1.296) | 0.670 |  |  |

*Abbreviations:* SII, systemic-immune-inflammation index; OR, odds ratio; CI, confidence interval; BMI, body mass index; COPD, chronic obstructive pulmonary disease; CKD, chronic kidney disease; ASA, American Society of Anesthesiologists physical status classification system; E.N.T, otolaryngology head, and neck surgery; SBP, systolic blood pressure; MAP, mean arterial pressure; WBC, white blood cell; ESR, erythrocyte sedimentation rate; Glu, glucose; Cre, creatinine; AST, aspartate aminotransferase; ALT, alanine aminotransferase; NSAIDs, non-steroidal anti-inflammatory drugs

**Table A4 Univariate logistic regression analyses for POD in the Model PSM.**

| **Variables** | **Univariate analysis** | |
| --- | --- | --- |
|  | **OR (95%CI)** | ***P*** |
| **SII (≤650 vs >650)** | 1.301(1.062-1.598) | 0.011 |
| **Age** | 1.089(1.073-1.106) | <0.001 |
| **Male (vs female)** | 1.147(0.935-1.409) | 0.19 |
| **BMI** | 0.962(0.935-0.99) | 0.009 |
| **Smoke (yes vs no)** | 1.081(0.855-1.355) | 0.509 |
| **Alcohol (yes vs no)** | 1.047(0.821-1.322) | 0.706 |
| **Hypertension (yes vs no)** | 1.138(0.929-1.394) | 0.212 |
| **Diabetes (yes vs no)** | 1.273(1.015-1.585) | 0.034 |
| **Cardiovascular diseases (yes vs no)** | 1.305(0.946-1.76) | 0.092 |
| **COPD (yes vs no)** | 1.82(1.23-2.604) | 0.002 |
| **Cerebrovascular disease (yes vs no)** | 1.739(1.309-2.275) | <0.001 |
| **CKD (yes vs no)** | 1.649(0.774-3.079) | 0.151 |
| **Depression and anxiety (yes vs no)** | 2.841(1.177-5.831) | 0.009 |
| **Non-independent functional status (yes vs no)** | 1.57(1.281-1.924) | <0.001 |
| **ASA (vs I)** |  |  |
| **II** | 0.926(0.345-3.79) | 0.897 |
| **III** | 2.141(0.792-8.785) | 0.199 |
| **IV** | 8.579(2.856-37.087) | <0.001 |
| **Days in hospital before surgery** | 1.012(1.003-1.024) | 0.018 |
| **Emergency surgery, (yes vs no)** | 2.828(1.912-4.052) | <0.001 |
| **Hemoglobin** | 0.983(0.978-0.988) | <0.001 |
| **WBC count** | 1.041(1.017-1.067) | <0.001 |
| **Monocytes** | 1.308(0.16-2.873) | 0.589 |
| **Glu** | 1.046(0.994-1.096) | 0.072 |
| **Albumin** | 0.903(0.882-0.924) | <0.001 |
| **Cre** | 1.003(1-1.005) | 0.018 |
| **Total bilirubin** | 1.002(0.999-1.004) | 0.109 |
| **AST** | 1(0.997-1.002) | 0.738 |
| **ALT** | 1.002(0.999-1.004) | 0.207 |
| **PT** | 1.222(1.142-1.306) | <0.001 |
| **Preoperative medication** |  |  |
| **Anticholinergic drug (yes vs no)** | 0.916(0.748-1.123) | 0.398 |
| **NSAIDs (yes vs no)** | 0.809(0.508-1.223) | 0.343 |
| **Benzodiazepines (yes vs no)** | 1.144(0.898-1.445) | 0.267 |
| **Antipsychotic drugs (yes vs no)** | 71.168(38.15-140.968) | <0.001 |
| **Type of surgery (vs Hepatopancreatobiliary and gastrointestinal surgery)** |  |  |
| **Orthopedic surgery** | 0.835(0.657-1.057) | 0.137 |
| **Urinary surgery** | 0.652(0.396-1.015) | 0.073 |
| **Thoracic surgery** | 0.472(0.261-0.788) | 0.007 |
| **E.N.T** | 0.613(0.338-1.026) | 0.082 |
| **Gynecology** | 0.742(0.364-1.346) | 0.367 |
| **Vascular surgery** | 1.28(0.741-2.072) | 0.344 |
| **Others** | 0.388(0.197-0.682) | 0.002 |
| **Anesthesia method (vs Basal anesthesia)** |  |  |
| **Epidural anesthesia** | 0.619(0.12-2.855) | 0.534 |
| **Nerve blocks** | 2.03(0.71-7.278) | 0.221 |
| **General anesthesia** | 1.28(0.535-4.188) | 0.629 |
| **General anesthesia combined with other anesthesia** | 1.227(0.488-4.123) | 0.699 |
| **Duration of surgery** | 1.004(1.003-1.004) | <0.001 |
| **Duration of anesthesia** | 1.003(1.002-1.004) | <0.001 |
| **Blood loss** | 1(1-1.001) | <0.001 |
| **Urine** | 1.001(1-1.001) | <0.001 |
| **Crystalloid** | 1(1-1) | <0.001 |
| **Colloid** | 1.001(1.001-1.001) | <0.001 |
| **Blood transfusion** | 2.311(1.851-2.871) | <0.001 |
| **Duration of SBP>140 mmHg** | 1.006(1.004-1.009) | <0.001 |
| **Duration of MAP<60 mmHg** | 1.013(1.009-1.017) | <0.001 |
| **Intraoperative medication** |  |  |
| **Glucocorticoid** | 1.143(0.924-1.42) | 0.223 |
| **Dexmedetomidine** | 1.104(0.782-1.517) | 0.559 |

*Abbreviations:* SII, systemic-immune-inflammation index; OR, odds ratio; CI, confidence interval; BMI, body mass index; COPD, chronic obstructive pulmonary disease; CKD, chronic kidney disease; ASA, American Society of Anesthesiologists physical status classification system; E.N.T, otolaryngology head and neck surgery; SBP, systolic blood pressure; MAP, mean arterial pressure; WBC, white blood cell; ESR, erythrocyte sedimentation rate; Glu, glucose; Cre, creatinine; AST, aspartate aminotransferase; ALT, alanine aminotransferase; NSAIDs, non-steroidal anti-inflammatory drugs.
